# Exposure to animals and the risk of allergic asthma: a population-based cross-sectional study in Finnish and Russian children

**DOI:** 10.1186/1476-069X-7-28

**Published:** 2008-06-06

**Authors:** Timo T Hugg, Maritta S Jaakkola, Risto Ruotsalainen, Vadim Pushkarev, Jouni JK Jaakkola

**Affiliations:** 1 South Karelia Allergy and Environment Institute, Joutseno, Finland and Institute of Health Sciences, University of Oulu, Oulu, Finland; 2 Institute of Occupational and Environmental Medicine, The University of Birmingham, Edgbaston, Birmingham B15 2TT, UK; 3 Department of Respiratory Medicine, Division of Medicine, University of Oulu, Oulu, Finland; 4 Director of the Indoor Air Quality Clinic, Allergy and Asthma Union, Paciuksenkatu 19, 00270 Helsinki, Finland; 5 Municipal Hospital of Svetogorsk, Pogranichnaya str. 13, Svetogorsk, Leningradskaya oblast, 188990, Russia; 6 Institute of Health Sciences, University of Oulu, Oulu, Finland

## Abstract

**Background:**

There is little information on potential differences in animal exposure between Finland and Russia and particularly on the effects of animal exposure on asthma among Russian children. The aim of the study was to compare the pet and farm animal exposures and to assess the relations of pre- and postnatal animal exposures to the occurrence of allergic asthma in Finnish and Russian school children.

**Methods:**

We conducted a population-based cross-sectional study in neighbour towns on either side of the Finnish-Russian border; Imatra in Finland and Svetogorsk in Russia. The study population consisted of 512 Finnish and 581 Russian school children aged 7–16 years (response rate 79%). Multivariate logistic regression analysis was used to estimate adjusted odds ratios (OR) and 95% confidence intervals (CI) related to each exposure.

**Results:**

Current indoor exposure to pets was more frequent among school children in Svetogorsk than in Imatra (67.5% vs. 56.0%, P < 0.001). Finnish children were exposed more frequently to dogs, whereas Russian children to cats during childhood and to farm animals during pregnancy and infancy. The risk of self-reported allergic asthma was inversely related to indoor dog keeping ever in Finland (adjusted OR 0.35, 95% CI 0.13, 0.95), whereas in Russia the risk of allergic asthma was increased in relation to combined indoor cat exposure during infancy and currently (4.56, 1.10, 18.91). The risk of asthma was elevated in relation to contact to farm animals during pregnancy (Finland: 1.95, 0.69, 5.50; Russia: 1.90, 0.70, 5.17) and early life (Finland: 2.05, 0.78, 5.40; Russia: 1.21, 0.39, 3.73).

**Conclusion:**

Exposure to pets and farm animals during childhood differed significantly between Finland and Russia. Our study provides evidence that early-life exposure to cats increases the risk of asthma whereas exposure to dogs is protective. Our findings suggest that intermittent fetal and early-life exposure to farm animals increases the risk of allergic asthma in urban children visiting farms.

## Background

In Western countries between 50% and 80% of households have pets [1,2]. Animal allergens can be detected in both public places and private facilities even without direct exposure to animals, so practically everyone has some exposure to animal allergens [3-5]. Although animal dander and animal secretions, such as saliva and urine, have long been recognized as being major allergens, studies on exposure to animals and asthma have provided somewhat conflicting results. Exposure to animals has been reported to both increase the risk of asthma [6,7] and diminish it [8,9].

As most people in the world are exposed to domestic animals in their residential environments, it is of importance to study the occurrence of these potentially adverse exposures under different regional and environmental conditions and to investigate the impact of different exposure patterns on the occurrence of allergic diseases. A number of studies on asthma in relation to exposure to animals have been reported [10-13], but only a few have examined national differences in childhood exposures [14,15]. We were able to identify only two previous studies that had compared animal exposures in Finland and Russia [16,17]. To our knowledge, this is the first study on the effects of early intermittent contact with farm animals on development of allergic asthma among urban populations, while previous studies have compared children living in farms to those not living in farms. This is also the first study on the effects of animal exposures in general on development of asthma comparing Finland and Russia, Russia being in the middle of processes of "westernisation" of lifestyle. The aim of the study was to compare exposure to animals in Finnish and Russian children and to assess the independent and combined effects of exposure to animals prenatally, postnatally and at the time of the study in school age on the risk of allergic asthma. The children were from Imatra, Finland and Svetogorsk, Russia, which are neighbour towns on either side of the Finnish-Russian border. The distance between the two study areas is only ten kilometres, but there are large differences in cultural habits, socio-economic conditions and health care system.

## Methods

### Study population

**Figure 1:**
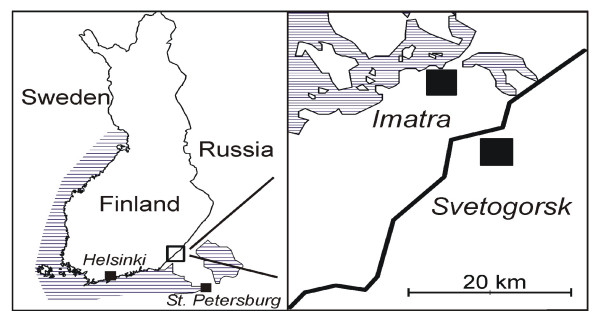
Map of the study area.

A cross-sectional, population-based study was conducted in the towns of Imatra, Finland and Svetogorsk, Russia, in October-November 2003. Imatra has a population of 30,000 and is located in the south-eastern part of Finland. Svetogorsk has a population of 17,000 and is located in the westernmost parts of the Saint Petersburg region. Both study areas are located in the border districts of the two countries (Figure 1). The study was approved by the local education and health authorities of both towns as part of a municipal co-operation project called "Twin City".

The source population consisted of all school children in the two municipalities. Participating primary schools, altogether four in Imatra and two in Svetogorsk, were selected so that the schools were representative of the area and populations of interest. All primary schools in Vuoksenniska district, Imatra, and all primary schools located in the town centre of Svetogorsk were invited to participate. In both countries the schools were closely surrounded by pulp and paper mills. Practically all school children both in Imatra (and in Finland in general) and Svetogorsk go to the school according to their place of residence. School children aged 7–16 years were asked to participate and those who returned a completed questionnaire formed the study population.

### Questionnaire

A self-administered questionnaire modified from the International Study of Asthma and Allergies in Childhood (ISAAC, Phases One, Two and Three) and Finnish questionnaires from two previous studies were distributed through schoolteachers to 1,400 school children aged 7–16 years. These questionnaires have been tested and validated [18-22]. The questionnaires were filled in anonymously at home by the school children, together with their parents, and returned by the child to his/her teacher. The Finnish questionnaire was translated into Russian by a Finnish-Russian certified translator assisted by a Russian medical expert. Questionnaires were distributed and collected so that no personal information of the participating individual could be identified.

### Health outcome and exposure assessment

The data on allergic asthma was obtained from the questionnaires using the following questions: Does your child have any allergies?" (no/yes), "If yes, does your child have asthma?" (no/yes). Presence of allergic asthma was defined as a confirmative answer to both of the question.

The assessment of exposure was based on exposure to animals as reported in the questionnaires, using the following questions: "Do you keep pets indoors in your home?" (no/yes); "What kind of pet(s) and how many is/are kept in the home"?; "How many years have you had pet(s) indoors?"; "Did you have cat(s)/dog(s) in your home during the child's first year of life?" (no/yes); "In your child's first year of life, did he/she have regular contact (at least once a week) with farm animals (e.g. cattle, pigs, horses, sheep or poultry)?" (no/yes); "Did the child's mother have regular contact (at least once a week) with farm animals (e.g. cattle, pigs, horses, sheep or poultry) while pregnant with this child?" (no/yes).

The following indices of exposure were formed to characterise exposure of the child to animals: exposure to farm animals during pregnancy (prenatal exposure), exposure to pets or farm animals during the child's first year of life (early-life exposure) and exposure to pets at the time of the study in school age (current exposure). The reference group consisted of subjects who had never owned a pet or had regular exposure to farm animals.

### Statistical methods

First, we compared the prevalences of exposure to animals between Finnish and Russian children, using X^2^- and T-tests for assessing statistical significance of the differences between the groups. Second, we assessed the relations of exposure to animals pre- and postnatally and at the time of the study to occurrence of asthma, using odds ratio (OR) as the measure of effect. Multivariate logistic regression analysis was used to estimate adjusted ORs and 95 % confidence intervals (CI) related to each exposure. Adjustment was made for the following core covariates: age, gender, moisture or visible mold at home, and parental smoking. Having ever had dogs, cats or exposure to farm animals were used as covariates when independent effects of each type of animal exposure were studied. Statistical analyses were conducted using the statistical package SAS, version 9.1 (SAS Institute, Cary, NC, USA).

## Results

### Study population

A total of 1,106 (response rate 79.0 %) children returned the questionnaire, 519 (74.1%) in Imatra and 587 (83.9%) in Svetogorsk. After excluding 13 subjects with incomplete data, the final study population consisted of 1,093 children; 512 from Finland and 581 from Russia. In Finland, 265 (51.8%) respondents were boys and 247 (48.2%) girls. In Russia, the corresponding numbers were 252 (43.4%) and 329 (56.6%), respectively. A total of 449 (88.7%) of the Finnish children lived in the town center or housing estate, 24 (4.7%) in a densely populated area and 33 (6.5%) in scattered settlements, whereas almost all the Russian children, 572 (99.5%) lived in the town center or housing estate and 3 (0.5 %) children lived in a densely populated area outside the town center. The prevalence of maternal (5.6% vs. 2.2%, P < 0.001) and paternal asthma (6.1% vs. 0.9%, P < 0.001) was higher in Finland compared with Russia. It is possible, but not very likely, that some of the 33 Finnish children living in scattered settlements could have had small-scale animal keeping at home.

### Animal exposures and allergic asthma

Table 1 shows that exposure to pets in general, and exposure to cats, birds and contact to farm animals were more common among Russian children, whereas exposure to dogs was more common among Finnish children. There was no difference in the mean duration of current indoor pet keeping between Finland (3 years) and Russia (3 years). Due to small number of pets in some subcategories, only most common groups/species (dog, cat, rodent and birds) were the focus of the further analyses.

**Table 1:** Self-reported animal exposure among school children in Imatra, Finland, and in Svetogorsk, Russia.

Exposure	Imatra (ntot = 512) % (n)	Svetogorsk (ntot = 581) % (n)	P value ^1^
Current indoor exposure to pets^2^	55.7 (285)	67.1 (390)	<0.001
*Indoor exposure to dogs *(≥ 1)^3^	26.4 (135)	16.0 (93)	<0.001
*Indoor exposure to cats *(≥ 1)^3^	20.9 (107)	45.6 (265)	<0.001
*Indoor exposure to rodents *(≥ 1)^3^	7.2 (37)	6.7 (39)	0.739
*Indoor exposure to birds *(≥ 1)^3^	2.7 (14)	7.8 (45)	<0.001
			
Exposure during child's first year of life			
*Indoor exposure to dogs or cats*	31.8 (163)^3^	27.2 (158)^2^	0.093
*Indoor exposure to dogs*^3^	12.7 (65)	3.1 (18)	<0.001
*Indoor exposure to cats*^3^	9.0 (46)	7.6 (44)	0.397
			
Regular contact with farm animals^2^			
*During pregnancy*	11.7 (60)	16.9 (98)	0.016
*During child's first year of life*	10.6 (54)	15.2 (88)	0.024

^1^P value, X^2 ^test

^2 ^Numbers are based on data available for the respective variable. For none of the variables did the missing data exceed 5%.

^3 ^Numbers are based on data available for the respective variable. For none of the variables did the missing data exceed 5%.

Because the animal exposure patterns differed substantially between Finland and Russia, the results are presented separately for the Finnish and Russian children (Tables 2 and 3). This was also done to get comparable groups when investigating the relations between animal exposure to asthma (i.e. comparing the exposed to the unexposed), even if it was likely that housing conditions and diagnostic practices differed to some extent between Finland and Russia. As a consequence of the smaller number of respondents in these subcategories, the estimates for some exposures have wide confidence intervals and relative risks related to some exposure groups could not be analysed in Russian children.

**Table 2:** Exposure to pets and farm animals and self-reported asthma in Finnish children (N = 512).

**Exposure indicators**	**Crude Odds Ratio**	**95% CI**	**Adjusted ^**1 **^Odds Ratio**	**95% CI**
*Current pets *(yes vs. no)				
Any pets indoors	0.56	0.27, 1.14	0.62	0.29, 1.31
Dog(s)	0.37	0.13, 1.06	0.37	0.13, 1.10
Cats(s)	0.36	0.11, 1.21	0.47	0.14, 1.58
Rodent(s)	Na^2^			
Bird(s)	1.12	0.14, 8.84	1.08	0.13, 9.27
*First year of life *(yes vs. no)				
Any pets indoors	0.56	0.22, 1.42	0.50	0.19, 1.30
Dog(s) indoors	0.57	0.08, 4.23	0.37	0.04, 3.18
Cat(s) indoors	1.25	0.17, 9.23	1.90	0.23, 16.07
*Dogs indoors*				
Never (reference)	1.00			
First year+; currently-	0.37	0.05, 2.78	0.36	0.05, 2.77
First year-; currently+	0.34	0.10, 1.15	0.37	0.11, 1.26
First year and currently	0.35	0.05, 2.67	0.31	0.04, 2.43
Any	0.35	0.13, 0.93	0.35	0.13, 0.95
*Regular contacts to farm animals*				
During pregnancy	2.10	0.82, 5.41	1.95	0.69, 5.50
First year of life	2.48	1.02, 6.01	2.05	0.78, 5.40
During pregnancy or first year	2.28	0.98, 5.29	2.00	0.81, 4.98

^1 ^Logistic regression analysis: adjusted for age, gender, parental smoking, and any dampness or visible mold.

^2 ^Na, not analysed: 0 asthma cases observed (2.6 expected) among 37 children with current exposure to rodents

**Table 3:** Exposure to pets and farm animals and self-reported asthma in Russian children (N = 581).

**Exposure indicators**	**Crude Odds Ratio**	**95% CI**	**Adjusted ^**1 **^Odds Ratio**	**95% CI**
*Current pets *(yes vs. no)				
Any pets indoors	0.57	0.24, 1.33	0.67	0.27, 1.65
Dog(s)	Na^3^			
Cats(s)	1.45	0.62, 3.42	1.65	0.67, 4.05
Rodent(s)	Na^4^			
Bird(s)	Na^5^			
*First year of life *(yes vs. no)				
Any pets indoors	1.28	0.51, 3.23	1.36	0.53, 3.54
Dog(s) indoors	Na^6^			
Cat(s) indoors	Na^7^			
*Cats indoors*				
Never (reference)	1.00			
First year+; currently-	Na^7^			
First year-; Currently+	1.15	0.46, 2.86	1.28	0.49, 3.34
First year and currently	3.48	0.90, 13.45	4.56	1.10, 18.91
Any	1.29	0.55, 3.04	1.46	0.60, 3.59
*Regular contacts to farm animals*				
During pregnancy	1.97	0.75, 5.22	1.90	0.70, 5.17
First year of life	1.26	0.42, 3.81	1.21	0.39, 3.73
During pregnancy or first year	1.64	0.65, 4.10	1.66	0.65, 4.27

^1 ^Logistic regression analysis: adjusted for age, gender, and any dampness or visible molds

^2 ^Logistic regression analysis: adjusted for age, gender, parental smoking, any dampness or visible mold, cats ever and farm animals ever.

^3 ^Na, not analysed: 0 asthma cases observed (4.2 expected) among 93 children with current exposure to dogs

^4 ^Na, not analysed: 0 asthma cases observed (1.6 expected) among 39 children with current exposure to rodents

^5 ^Na, not analysed: 0 asthma cases observed (1.8 expected) among 45 children with current exposure to birds

^6 ^Na, not analysed: 0 asthma cases observed (0.4 expected) among 11 children with early exposure to dogs

^7 ^Na, not analysed: 0 asthma cases observed (0.6 expected) among 16 children with early exposure to cats

The prevalence of self-reported allergic asthma was higher in Imatra than in Svetogorsk (6.7% vs. 3.9%, P = 0.047, respectively). Correspondingly the prevalence of wheezing was higher in Imatra than in Svetogorsk (7.6% vs. 6.6%, P = 0.550, respectively) during the last 12 months. In Finnish children in general, the risk of asthma was inversely related, although mostly not significantly so, to exposure to pets during the first year of life and at the time of the study. As an exception to this trend, exposure to cats in the first year of life was related to somewhat increased risk (Table 2). The relation between having ever kept a dog indoors and allergic asthma showed a statistically significant protective effect (adjusted OR 0.35, 95% CI 0.13, 0.95). This effect estimate remained unchanged after additional adjustment for having ever had cats or contact to farm animals. On the other hand, regular contact with farm animals during the first year of life and during pregnancy or first year of life was related, although not significantly so, to increased risk of allergic asthma (adjusted OR 2.05, 95% CI 0.78, 5.40 and 2.10, 0.83, 5.35, the latter being adjusted also for exposure to pets, respectively).

In Russian children in general, childhood exposure to pets was associated, although mostly not significantly so, with an elevated risk of allergic asthma (Table 3). Exposure to cats both during the first year of life and at the time of the study was related to a significantly increased risk of allergic asthma (adjusted OR 4.56, 95% CI 1.10, 18.91). There were no cases of asthma among the 93 children exposed to dogs, which was consistent with the protective effect observed among Finnish children, as 4.2 cases of asthma would have been expected in this group based on occurrence of asthma in the unexposed group. The relation of asthma with regular exposure to farm animals was consistent with that observed in Finnish children: the adjusted OR of allergic asthma for farm animal exposure during pregnancy and during pregnancy or first year of life was 1.90 (95% CI 0.70, 5.17) and 1.71 (0.67, 4.41, adjusted also for exposure to pets), respectively.

## Discussion

The pattern of exposure to animals differed substantially between Finnish and Russian children. The Russian children were more commonly exposed to cats, birds and farm animals, while dog exposure was more common in Finnish children. In Finnish children, the risk of allergic asthma was decreased among children exposed to dogs, but there was some indication that cat exposure during the first year of life increases the risk of allergic asthma. In Russian children the finding of no cases of asthma among children exposed to dogs was consistent with a protective effect. In contrast, exposure to cats during the first year of life or at the time of the study increased the risk of asthma among Russian children. The risk of allergic asthma was also elevated in relation to regular contact with farm animals consistently both in Finnish and Russian children.

### Validity of results

The response rate was good in both study areas (74% and 84%), giving us assurance that the study population reflected the school-age child populations of these areas rather well.

Exposure assessed based on current pet keeping may be influenced by pet avoidance behaviour by allergic families. In some previous studies, 1.7–27.3% of respondents reported avoiding contact with pets because of allergy [7,9] and 4.7–12% of respondents gave up pets because of allergy [23,24]. On the other hand, Svanes et al. [24] observed that parents who kept pets despite their own asthma did so even if their child got asthma, and adults who already had a pet at the time of developing an allergic disease continued to keep it despite the allergy. In this study, there was a difference in the prevalence of pet ownership at the time of the study between asthmatic and non-asthmatic children (in Finland: 42.4% and 56.6% had currently pet(s), respectively; in Russia: 54.6% and 67.6%, respectively) and by having asthmatic and non-asthmatic parents or siblings (in Finland: 47.7% and 57.3% had currently pets, respectively; in Russia: 63.0% and 67.3%, respectively). This suggests that some pet avoidance behaviour may have taken place, although we did not ask about pet avoidance directly.

The relation between a pet and a family is generally so close and established that there is little reason to believe that many individuals would have trouble recalling information on pet ownership [6,24]. In addition, Almqvist et al. [25] have shown good consistency between questionnaire data and objective measurements of allergen levels.

Due to the likelihood of national differences in diagnostic procedures and definitions of asthma [26-28], self-reported allergic asthma was used instead of doctor-diagnosed asthma. Asthma was traditionally defined in Soviet Union/Russia as an allergic or atopic disease including such symptoms and signs as bronchospasm, dyspnoea, and hypersecretion and swelling of the bronchial mucosa. The concept 'infectious allergy' was also used and asthma was even considered as a complication or a subgroup of chronic bronchitis and pneumonia [26]. In contrast in Finland, occurrence of nocturnal cough, dyspnoea during or after exercise, and/or wheezing together with reversibility in spirometric lung functioning tests (PEF, FEV_1_, and histamine or metacholine challenge tests) indicating variable bronchial obstruction have been used as diagnostic criteria for asthma [29]. However, in this study the prevalence of self-reported asthma was well in line with the prevalence of self-reported doctor-diagnosed asthma. The difference in the prevalence based on self-reported doctor-diagnosed and self-reported asthma was small, from 0.2% (asthma in Finland) to 0.5% (asthma in Russia), and use of doctor-diagnosed asthma in additional analyses did not lead to any notable changes in the observed relations when compared to the results reported here for self-reported asthma. Occurrence of wheezing was higher than the prevalence of self-reported asthma, which is no surprise as wheezing is less specific than asthma, but these differences in prevalence may indicate a tendency to underdiagnose asthma, particularly in Russia.

To control potential confounding, we adjusted for the following core covariates: age, gender, moisture or visible mold at home, and parental smoking. Parental atopy and allergies are known determinants of the child's asthma [30] and were associated with pet keeping, thus satisfying two main criteria for a potential confounder. However, pet keeping could increase the risk of parental asthma [31] and therefore pet keeping could be in the causal pathway of the relation between pet exposure and childhood asthma. Because of this, we did not adjust for parental atopy or allergies in the final models. In additional analyses performed, inclusion of parental asthma/allergy had little influence on the effect estimates.

### Synthesis with previous knowledge

Our findings suggest that continuous home exposure to cat allergens increases the risk of self-reported allergic asthma, whereas exposure to dogs decreases the risk. Findings from both Finnish and Russian children are consistent, although not statistically significantly so, with the adverse effect related to intermittent early farm animal exposure.

The prevalence of pet exposure in the present study was relatively high, being in line with the results of previous studies from other countries [7,10,24]. The results are also in accordance with results of recently published studies from Finland and Russia [16,32,33]. According to Gusareva et al. [32] cat is a major allergen among west Siberian patients with asthma. Interestingly, indoor exposure to cats during child's first year of life was, although not significantly so, lower in Russia than in Finland, although current keeping of cat(s) indoors was twice as common in Russia as in Finland. These results indicate that (indoor) cat keeping is a relatively new phenomenon and/or that acquisition of a pet occurs mostly later in life in Russia. In contrast to the suggestion of Al-Mousawi et al. [13], our results from Russia indicate that even in areas with relatively high prevalence of cats, continuous indoor exposure to cats can increase the risk of asthma. In line with the present Finnish results, Hesselmar et al. [10] and Hölscher et al. [8] have observed that pet exposure during the first year of life or currently is inversely associated with childhood asthma.

There is some previous evidence that exposure to dogs and cats may have different effects on the risk of asthma. Linneberg et al. [34] found that previous or continuing exposure to a cat at home increased the risk of developing a sensitization to cat in adulthood, whilst having a dog at home did not increase the risk of developing a sensitization to dog. Similarly, Oberle et al. [35] observed a significant association between continuous exposure to cats from early life on and asthma in childhood, whereas exposure to dogs was not related to the prevalence of asthma. In addition, some previous studies have suggested that cat allergens could be more potent in causing sensitisation than dog allergens [36,37].

To our knowledge, there is no previously published data on the prevalence of farm animal exposure during pregnancy and child's first year of life among Russian school children. However, in line with the present study, von Hertzen et al. [16] showed that frequent contacts with farm animals were more common among Russian than among Finnish school children during the last 12 months. In previous studies, lower frequencies of asthma have been observed in children growing up on farms compared to those reared in non-farm areas [38,39]. However, little has been known about the risk of asthma among children with urban background having regular contact with farm animals during childhood. In the present study, the risk of asthma was elevated in relation to contact with farm animals during pregnancy and early life both in Finnish and Russian children who did not live in the farms. It seems reasonable to speculate that the protective effects of animal exposure are allergen specific [40,41] and dose dependent [42]. It is possible that regular but intermittent pre- and postnatal exposure to farm animals is not intensive enough to cause protective response among children with urban background. Alternatively, urban children exposed to farm animals only during visits may not experience some of the environmental or dietary exposures that may be typical for children growing up in farms that are protective against allergies [42].

Gereda et al. [43] stated that households with detectable allergen levels but low levels of certain microbial products (mainly endotoxins) may provide an environment that predisposes for animal allergen sensitisation. As the level of endotoxins is related to the presence or absence of animals, this could be a step in the causal pathway, so should not be adjusted for in the models. Different microbial load in general between Russia and Finland could modify the relations, which is one reason why we performed analyses in Russian and Finnish children separately. Unfortunately, we did not collect any information on endotoxins in this study, but we did adjust for indoor dampness and mold problems which partly adjusts for microbial exposures.

## Conclusion

Our results show that exposure to animals differ between Russian and Finnish school children. Our study provides evidence that early-life exposure to cats increases the risk of allergic asthma whereas exposure to dogs seems to be protective. Our findings suggest that intermittent fetal and early-life exposure to farm animals increases the risk of allergic asthma in urban children visiting farms.

## Competing interests

The authors declare that they have no competing interests.

## Authors' contributions

TH conceived of the study, participated in the design of the study, performed partly the statistical analysis and wrote the manuscript, MJ participated in the design and coordination of the study and helped to draft and revise the manuscript, RR participated in the design and coordination of the study, VP participated in the design, coordination and acquisition of the study, JJ participated in the design and coordination of the study, performed the majority of the statistical analysis, helped to draft the manuscript and gave final approval of the version to be published. All authors read and approved the final manuscript.
